# Autophagy-Associated Immunogenic Modulation and Its Applications in Cancer Therapy

**DOI:** 10.3390/cells11152324

**Published:** 2022-07-28

**Authors:** Zhuxi Duan, Yu Shi, Qun Lin, Ahmed Hamaï, Maryam Mehrpour, Chang Gong

**Affiliations:** 1 Guangdong Provincial Key Laboratory of Malignant Tumor Epigenetics and Gene Regulation, Sun Yat-Sen Memorial Hospital, Sun Yat-Sen University, Guangzhou 510120, China; duanzhx5@mail2.sysu.edu.cn (Z.D.); shiy77@mail2.sysu.edu.cn (Y.S.); linq27@mail2.sysu.edu.cn (Q.L.); 2 Breast Tumor Center, Sun Yat-Sen Memorial Hospital, Sun Yat-Sen University, Guangzhou 510120, China; 3 Institut Necker-Enfants Malades (INEM), Inserm U1151-CNRS UMR 8253, Université Paris Descartes-Sorbonne Paris Cité, F-75993 Paris, France; ahmed.hamai@inserm.fr (A.H.); maryam.mehrpour@inserm.fr (M.M.)

**Keywords:** autophagy, immunity, cancer therapy

## Abstract

Autophagy, a lysosome-mediated cellular degradation pathway, recycles intracellular components to maintain metabolic balance and survival. Autophagy plays an important role in tumor immunotherapy as a “double-edged sword” that can both promote and inhibit tumor progression. Autophagy acts on innate and adaptive immunity and interacts with immune cells to modulate tumor immunotherapy. The discovery of autophagy inducers and autophagy inhibitors also provides new insights for clinical anti-tumor therapy. However, there are also difficulties in the application of autophagy-related regulators, such as low bioavailability and the lack of efficient selectivity. This review focuses on autophagy-related immunogenic regulation and its application in cancer therapy.

## 1. Introduction

**Figure 1:**
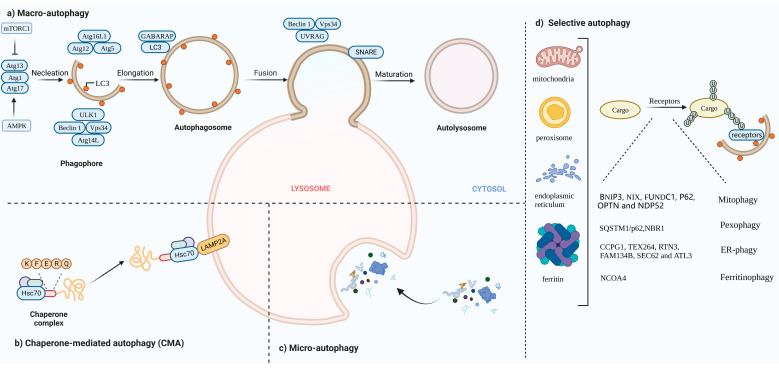
(**a**) Macro-autophagy: The occurrence of autophagy mainly involves five major processes: (1) Autophagy induction phase. In this phase, mammalian target of rapamycin complex 1(mTORC1) activity is inhibited; ATG13 phosphorylation is reduced; and the ATG13–ATG1–ATG17 complex is formed. (2) Vesicle nucleation. The Vps34–Beclin1 complex mediates the formation of pre-autophagosomal structure (PAS). (3) The elongation stage of the autophagosome. During this phase, the ATG12–ATG5–ATG16 complex is assembled and microtubule-associated protein light chain3 (LC3) LC3 is converted from the water soluble form (LC3 I) to a fat soluble form (LC3 II). (4) Random capturing or selective targeting for degradation. (5) Autophagosomes fuse with lysosomes and autophagosome cleavage. (**b**) CMA: Hsc70 specifically mediates protein degradation via receptor LAMP2A. (**c**) Micro-autophagy: Lysosomes directly engulf aggregates. (**d**) Selective autophagy: Specific intracellular components combine with individual cargo for autophagic degradation, including mitophagy, pexophagy, ER-phagy and ferritinophagy.

Autophagy, first discovered by Belgian chemist Christine in 1963, is a process that is responsible for transporting damaged organelles, misfolded proteins and other macro molecules to lysosomes for degradation and regeneration [1]. It is a phenomenon that widely exists in eukaryotic cells. Several studies have shown that autophagy is triggered to varying degrees by processes such as angiogenesis and osteogenic differentiation during the differentiation of many cells [2,3,4]. Autophagy can be non-selective, when the cell is in an energy emergency to uptake generic cytoplasmic materials, or selective, to specifically remove damaged organelles, including mitochondria, ER, Golgi membranes and protein aggregates. The occurrence of autophagy mainly involves five major processes. In the autophagy induction phase, the counterbalanced control of mammalian target of rapamycin complex 1(mTORC1) and AMP-activated protein kinase (AMPK) via amino acid deprivation enhances ATG13 and unc-51-like autophagy-activating kinase 1 (ULK1) phosphorylation, and the ATG13–ATG1–ATG17 complex is formed. During vesicle nucleation, the lipid kinase activity of Vps34 facilitates the formation of a pre-autophagosomal structure (PAS), with Beclin 1 and ATG-14 like protein (ATG14L), inducing a ATG-conjugation cascade downstream. In the elongation stage of the autophagosome, the association of the ATG12–ATG5–ATG16 complex lapidates microtubule-associated protein light chain3 (LC3) or γ-aminobutyric acid receptor-associated protein (GABARAP) from the water-soluble form to a fat-soluble form. Lipidated LC3/GABARAP cooperates with other factors to elongate and close autophagosomes. WD repeat domain phosphoinositide-interacting protein 1 (WIPI 1) combines with Vps34-derived Ptdlns_3_phosphate (Ptdlns_3_P), cooperating with ATG2 and ATG9 to form autophagic organelles. During cargo assembling, LC3 II serves as a receptor for autophagy substrates, either to randomly capture or selectively target for degradation. For the final fusion with lysosomes, ultraviolet radiation resistance-associated gene protein (UVRAG) replaces ATG14L in the Vps34–Beclin 1 complex. The attachment of SNARE to the membrane of autophagosomes enables the fusion with lysosomes, following autolysosome degradation [5,6,7,8] (Figure 1a).

The tumor microenvironment (TME) is the surrounding environment in which the tumor grows and survives. It is now generally accepted that tumor cells, immune cells, stromal cells and the extracellular matrix (ECM) closely interact to form the main structure of the TME. These cells affect biological processes such as tumor growth and metastasis by secreting cytokines and releasing signaling molecules. Macrophages, lymphocytes, natural killer (NK) cells and dendritic cells (DCs), among the immune cells, are critical for tumor-cell killing and tumor control. However, some immunosuppressive cells, such as myeloid-derived suppressor cells (MDSCs), regulatory T cells (Tregs) and type 2 polarized macrophages (M2 macrophages), are also present in the tumor microenvironment to counteract the anticancer immune response [9,10]. Tumor immunotherapy, such as oncolytic-virus therapies, cancer vaccines, cytokine therapies, adoptive cell transfer and immune checkpoint inhibitors targeting the TME, has made rapid progress in recent years. There is growing evidence that autophagy can be involved in the regulation of the innate and adaptive immunity [11]. At the same time, some immune cells and cytokines can also affect and regulate autophagy. Autophagy plays an important role in tumor immunotherapy as a “double-edged sword” that can both promote and inhibit tumor progression.

This review discusses the classification and mechanism of autophagy, the relationship between autophagy and innate or adaptive immunity and the role of autophagy in anti-cancer immunotherapy.

## 2. The Landscape and Forms of Autophagy

Autophagy can be divided into three categories based on how substances are packaged and transported, including macro-autophagy, micro-autophagy and chaperone-mediated autophagy (CMA) [12]. Macro-autophagy is the most common form of autophagy. Cytosolic substrates are wrapped by endoplasmic reticulum or mitochondria-derived bilayer membranes to form autophagosomes. After fusion with lysosomes, the cargo is degraded, and the resulting macromolecules are released back into the cytoplasmic matrix for reuse [13]. Micro-autophagy is the invagination of the lysosomal membrane itself, encapsulating and phagocytosing the substrates to be degraded in the cell and degrading them in a lysosome. The difference between micro-autophagy and macro-autophagy is the absence of autophagosomes [14,15]. Chaperone-mediated autophagy, independent of vesicle trafficking, often relies on chaperone protein Hsc70 to specifically degrade target proteins with a unique recognition pentapeptide motif (KFERQ-like motif) and receptor protein LAMP2A on the lysosomal membrane to recognize the binding-protein complex. The KFERQ group “guides” the target protein into the lysosome for degradation [16,17] (Figure 1b,c).

In this review, we mainly focus on macro-autophagy (hereafter referred to as autophagy). Generally, autophagy has been regarded as a non-selective transport of cytoplasmic components to lysosomes for bulk degradation since it appears to indiscriminately engulf cytosol. However, autophagy may also be highly selective. Transmission electron microscopy has detected autophagic compartments with different contents in mammalian cells, including mitochondria, ER and Golgi membranes [18]. Selective autophagy typically occurs under nutrient-rich conditions to remove damaged or redundant organelles such as mitochondria, endoplasmic reticulum (ER) and Golgi complexes [19]. Moreover, it depends on soluble or membrane-bound selective autophagy receptors (SARs) to degrade the specific intracellular components. According to autophagosomes with different contents, selective autophagy can be divided into mitophagy, lipophagy, ER-phagy, ferritinophagy, etc.

### 2.1. Mitophagy

In yeast and mammalian cells, damaged mitochondria can be selectively degraded via mitophagy, regulating the number of mitochondria in cells and maintaining normal function [20]. There are two main pathways that mediate mitophagy.

#### 2.1.1. Receptor-Mediated Mitophagy

BNIP3, NIX, FUNDC1, PHB2. BNIP3, NIX and FUNDC1 receptors are localized to the OMM and directly interact with LC3 to mediate mitochondrial clearance [21,22,23]. NIX and Bnip3 promote the selective degradation of mitochondria during reticulocyte maturation [24,25]. The phosphorylation of BNIP3 and NIX enhances their interaction with LC3 [26]. After mitochondrial damaging, PHB2 and cardiolipin externalize to the OMM and interact with LC3 [27].

#### 2.1.2. Ubiquitin-Mediated Mitophagy

PINK1/Parkin pathway: Under stress conditions, auto-phosphorylated PINK1 promotes Parkin recruitment and also leads to Parkin activation and the ubiquitination of substrates on damaged mitochondria that function as autophagy-mediated degradation signals [28,29]. P62, OPTN and NDP52 [30] recognize phosphorylated polyubiquitin chains on mitochondrial proteins and initiate autophagosome formation by binding to LC3.

### 2.2. Pexophagy

Under conditions of nutrient starvation or ROS burst, peroxisomes are degraded in an autophagic manner to maintain cellular homeostasis [31]. In response to ROS, ataxia-telangiectasia mutated (ATM) interacts with peroxisomal signal-receiving molecule PEX5, translocating to the peroxisome surface [32]. Then, phosphorylated PEX5 is further ubiquitinated by PEX2/10/12 and binds to p62/NBR1, interacting with LC3 to promote the occurrence of autophagy [33,34].

### 2.3. ER-Phagy

The ER is the largest organelle in the cell and has functions such as folding, processing and transporting proteins, and regulating cellular metabolism [35]. When unfolded proteins accumulate on the ER, ER-phagy is activated to degrade damaged ER, inhibit protein synthesis, relieve ER stress and enable cell survival. Six receptors have been identified in mammals that respond to the ER, CCPG1, TEX264, RTN3, FAM134B, SEC62 and ATL3, which contain at least one critical LIR/GIM domain interacting with LC3 II (or ATG8)/GABARAP to mediate the occurrence of ER autophagy [36,37,38,39,40,41,42].

### 2.4. Ferritinophagy

In the presence of low iron concentration in cells, ferritin is degraded in lysosomes through the activation of ferritinophagy. Iron (Fe) is stored in a ferritin complex containing ferritin heavy chain (FTH1) and light chain (FTL). Nuclear receptor coactivator 4 (NCOA4) binds to ferritin and mediates its delivery to the autophagosome. The fusion of autophagosomes with lysosomes results in ferritin degradation and subsequent iron release [43,44] (Figure 1d).

## 3. The Relationship between Autophagy and Immunity

### 3.1. Innate Immunity and Autophagy

#### 3.1.1. Autophagy and Inflammation

**Figure 2:**
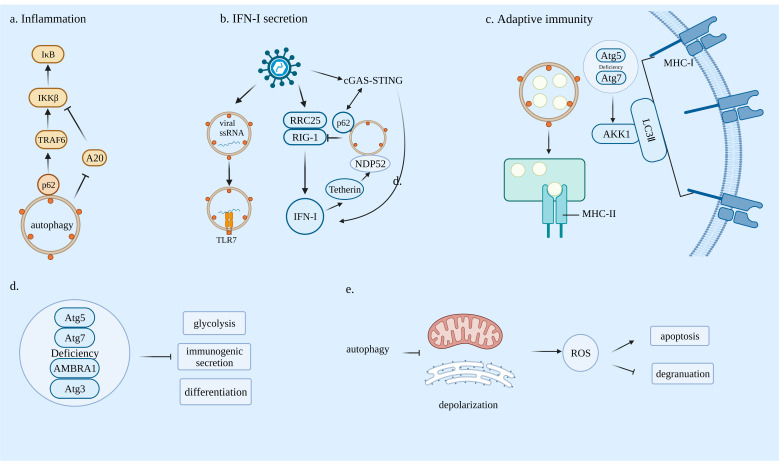
(**a**) The role of autophagy in the regulation of inflammation: Selective autophagy interacts with TNF receptor associated factor 6 (TRAF6) through p62 cargo, thus activating the NF-κB pathway via the IkappaB kinase β subunit (IKKβ) and IκB. It can also degrade A20, an NF-κB inhibitor, to enhance NF-κB activation. (**b**) Several examples of the role of autophagy in antiviral type I interferon (IFN-I) response: Upon virus infection, autophagy can deliver pathogen-associated molecular patterns (PAMPs) to cytosolic Toll-like receptors (TLRs) for their activation. P62-mediated autophagy inhibits retinoic acid-inducible gene I (RIG-I)-dependent IFN-I secretion. The cGAS–STING pathway inter-inhibitively interacts with autophagy to control IFN-I secretion. Tetherin, as an interferon-inducible antiviral factor, modulates NDP52-mediated selective autophagy. (**c**) The roles of autophagy in adaptive immunity: Autophagy can enhance intracellular antigen presentation via MHC-II. The deficiency of Atg5-Atg12 results in the increase in MHC-I through the accumulation of adapter protein-2 associated kinase 1 (AAK1). (**d**) The deletion of autophagy-related genes can dysregulate the vital functions of immune cells, including glycolysis, immunogenic secretion and differentiation. (**e**) Autophagy deficiency results in excessive reactive oxygen species (ROS) through mitochondria and endoplasmic reticulum (ER) accumulation, leading to immune-cell apoptosis and degranulation dysfunction.

In innate immunity, pathogen-associated molecular patterns (PAMPs) are recognized by Toll-like receptors (TLRs) on antigen presenting cells (APCs) and further TLRs recruit the MYD88 and IRAK families. Subsequently, activated IRAK promote the aggregation and ubiquitination of TRAF6 and further activate TAK1 and IKK. The ubiquitination of TRAF6 and NEMO promote the activation of the catalytic IKKβ subunit, leading to the phosphorylation and proteasomal degradation of IκB. Moreover, NF-κB heterodimers translocate into the nucleus and release proinflammatory cytokines. Selective autophagy plays an important role in the activation of the NF-κB signaling pathway. P62 through the TB motif selectively interacts with the TRAF domain of TRAF6 [45], which promotes TRAF6 oligomerization and ubiquitination and activates the NF-κB pathway. P62 has also been found to sequester A20 (an NF-κB inhibitor) in autophagosomes, which promotes macrophages to enhance NF-κB activation and release chemokines to recruit neutrophils [46]. It has also been found that under IL-1α stimulation, there is an autoregulatory loop whereby NF-κB regulates p62 expression, which in turn extends NF-κB activation in mouse and human pancreatic ductal adenocarcinoma (PDAC) cell lines [47] (Figure 2a).

TLR2 mediates macro-autophagy through the JNK [48] or ERK [49] signaling pathway. TLR4 regulates autophagy through a TRIF-dependent, MyD88-independent signaling pathway. RIP1 and p38 MAPK are downstream components of this pathway [50].

#### 3.1.2. Autophagy and Antiviral Type I Interferon Responses

Pattern recognition receptors (PRRs) including TLR RIG-like receptors (RLRs) and Nod-like proteins (NLRs) are an important part of the body’s innate immune system. They exist in various forms and are not only expressed on cell membranes but also widely distributed in endosomal membranes, lysosomal membranes and the cytoplasm. They identify pathogen-associated molecular patterns (PAMPs) and then activate the relevant anti-inflammatory pathways to induce the production of cytokines and interferons, thereby stimulating the body’s innate immune response. Autophagy mainly regulates the expression of IFN-I through PRR signaling pathways [51]. Lee et al., have found that TLR7 recognizes ssRNA requiring autophagy to transport cytosolic replication intermediates of viruses from the cytoplasm to lysosomes [52]. Du et al., have identified that during RNA virus infection, leucine-rich repeat containing protein 25 (RRC25) binds to ISG15-associated immune receptor (RIG-I) and regulates its degradation via p62-mediated selective autophagy, thereby limiting RIG-I-dependent IFN-I signaling [53]. Jin et al., have demonstrated that Tetherin (an interferon-inducible antiviral factor) recruits E3 ubiquitin ligase MARCH8 to catalyze K27-linked ubiquitin chains on MAVS at lysine 7, thereby modulating the NDP52-dependent selective autophagy pathway [54]. The cGAS–STING signaling pathway can induce IFN-I expression and participate in innate immune responses by recruiting and promoting TBK1 autophosphorylation under the trigger of cytoplasmic DNA, consequently activating the IRF3 transcriptional pathway, or by activating the NF-κB signaling pathway [55,56]. Prabakaran et al., have reported that upon DNA viral infection, the cGAS–STING pathway activates TBK 1, which can both phosphorylate IRF3 to induce IFN-I expression and phosphorylate p62. Activated p62 mediates selective autophagy, thereby promoting STING degradation and attenuating the cGAS–STING-pathway response [57] (Figure 2b).

### 3.2. Adaptive Immunity and Autophagy

Several studies have shown that autophagy is associated with T-cell-mediated cellular immune responses.

#### 3.2.1. Autophagy Can Affect Antigen Presentation

Previous studies have shown that autophagy promotes the MHC class II presentation of peptides from intracellular source proteins [58]. However, the inhibition of autophagy can also lead to increased antigen presentation. In ATG5- and ATG7-deficient dendritic cells (DCs) with reduced endocytosis and degradation, MHC class I on the surface increases due to the recruitment of internalization factor AAK1 via LC3 II lipidation [59]. Yamamoto et al., have found that in PDAC, MHC-I molecules are selectively targeted for lysosomal degradation through an autophagy-dependent mechanism involving the autophagy cargo receptor NBR1 which inhibits antigen presentation, reduces T cell responses and promotes immune evasion of pancreatic cancer [60,61] (Figure 2c).

#### 3.2.2. Autophagy Is Essential for Lymphocyte Development

Atg7-mediated mitophagy is indispensable for hematopoietic stem cell (HSC) maintenance [62]. The plasmacytoid dendritic cell (pDC) secretion of IFN-α requires autophagy [52]. Mitochondrial clearance is important during T-cell maturation, and autophagy helps to remove excess mitochondria from T cells. Autophagy-deficient T cells produce increased reactive oxygen species [63].

## 4. Autophagy-Associated Immunogenic Modulation in Tumor-Infiltrating Immune Cells

### 4.1. T Cells

Autophagy is required for T cells to maintain basic homeostasis. It may function as nutritional backup and immune–metabolic modulator to provide quality control.

#### 4.1.1. Naïve T

Autophagy is essential for peripheral-naïve-T-cell survival. Atg3-deficient naive CD4^+^ and CD8^+^ T cells have a defective survival [64]. The acute deletion of Atg3 does not result in a decrease in naïve-T-cell survival, whereas the accumulation of organelles such as mitochondria and ER results in death beyond 24 days. In spite of the fatal regulation of organelle homeostasis, the maturation of naïve T cells requires autophagy-dependent mitochondrial reduction. Autophagy can also promote the metabolic shift of T cells. The LIR motif of TAX1BP1 is thought to interact with the LC3 protein, activate selective autophagy and mTORC1, and conduct the metabolic shift of activated T cells [65].

#### 4.1.2. CD8^+^T

CD8^+^T is an important lymphocyte for the body to clear viral infection. Autophagy deficiency negatively affects CD8^+^T more than CD4^+^T [66]. Autophagy is a critical regulator of memory-CD8^+^T-cell formation. Mice lacking Atg7 in T cells fail to establish CD8^+^T-cell memory [67]. However, it has also been found that although the loss of Atg5 results in a marked reduction in the total number of CD8^+^Ts, it profoundly increases their transition to effector memory cells producing IFN-γ and TNF-α, thereby enhancing their antitumor activity [68].

#### 4.1.3. Tregs

Treg-cell-mediated antitumor immune suppression is the main mechanism of tumor immune escape [69]. Autophagy has been shown to be closely linked to Tregs [70]. The deletion of autophagy-related genes ATG5, ATG7 and AMBRA1 causes Treg dysfunction in mouse cells [71,72,73]. The defection of autophagy due to the lack of Atg5 or Atg7 can upregulate metabolic regulators mTORC1 and c-Myc, as well as glycolysis, resulting in Treg deficiency [71].

### 4.2. B Cells

Autophagy plays an important role in B-cell development. Atg5 is necessary for the maintenance of B-1a B-cell numbers and maintaining B-cell development [74]. The activation of autophagy is a mechanism for autoreactive B-cell survival. Moreover, Atg7-deficient B cells greatly reduce the ability to differentiate into plasma cells and the levels of immunoglobulin IgM secretion [75,76]. Tumor-derived autophagosomes (DRibbles)-induced B-cell activation can be enhanced by macrophages via CD40/CD40L. Moreover, the activation of macrophages is largely dependent on the TLR4 and MyD88 signaling pathways [77].

### 4.3. Macrophages

Tumor-associated macrophages (TAMs) are macrophages infiltrating in tumor tissues, mainly differentiated from monocytes. Chemokines such as CSF1 and CCL2 secreted by tumors can recruit monocytes in the peripheral blood to the tumor microenvironment (TME), following the differentiation from monocytes to TAMs [78].

#### 4.3.1. Autophagy Plays a Role in Monocyte or Macrophage Recruitment

CCL2 and IL-6 play important roles in the recruitment of monocytes to the TME. They can also promote monocytes differentiation into M2-type macrophages via the inhibition of caspase-8 cleavage and autophagy [79]. Recombinant capsid protein VP1 (rVP1) induces BECN1-dependent autophagy and enhances MAPK1/3 phosphorylation and MMP9 activity to promote macrophage migration [80].

#### 4.3.2. Autophagy Is Necessary for the Differentiation of Monocytes into Macrophages

Monocytes differentiating into macrophages is a caspase-dependent process triggered by colony stimulating factor1 (CSF-1). CSF-1 increases the expression and phosphorylation of ULK1, thereby contributing to the increased induction of autophagy. Moreover, in the absence of ATG7, the differentiation of monocytes into macrophages and the phagocytic ability of macrophages are severely impaired [81].

#### 4.3.3. Autophagy Is Highly Correlated with Polarization of Macrophages

Macrophages themselves are heterogeneous, and activated macrophages mainly include M1-type macrophages and M2-type macrophages. M1-type macrophages can kill tumor cells and resist pathogen invasion, while M2-type macrophages mainly play a role in promoting tumor growth, invasion and metastasis [82]. The polarization of M1-type and M2-type macrophages in the TME is dependent on the NF-κB pathway [83]. Hepatoma-derived TLR2 signaling induces the cytoplasmic ubiquitination of NF-κB RELA, leads to its degradation through p62/SQSTM1-mediated selective autophagy and further stimulates M2-type-macrophage differentiation [84].

### 4.4. Natural Killer Cells (NK Cells)

NK cells exert their antitumor effects by directly killing tumor cells, inducing apoptosis, secreting IFN-γ and inhibiting tumor metabolism [85]. Autophagy appears in immature NK (iNK) cells and is essential for NK cell development. Autophagy deficiency in NK cells leads to mitochondrial damage and accumulation of ROS, which ultimately leads to NK-cell apoptosis. Phosphorylated FOXO1 in the cytoplasm of iNK interacts with ATG7 to induce autophagy, thereby promoting NK-cell development and NK-cell-induced innate immunity [86]. Furthermore, BNIP3- and BNIP3L-mediated mitophagy promotes natural-killer-cell memory generation [87].

### 4.5. Neutrophils

Neutrophils, as the first line of defense against pathogens, are a very important part of the innate immune system and a major source of ROS production. Autophagy is involved in neutrophil differentiation. There are five stages of neutrophil differentiation: myeloblasts (MBs), myelocytes (MCs), metamyelocytes (MMs), band cells (BCs) and neutrophils (PMNs). ATG5 has been shown to be associated with neutrophil differentiation [88]. Autophagy provides free fatty acids by mediating lipolysis to support the mitochondrial respiration pathway, essential for neutrophil differentiation. The deficiency of ATG7 in neutrophil precursors results in increased glycolytic activity, whereas impaired mitochondrial respiration, reduces ATP production and stunts neutrophil differentiation [89]. Autophagy is also required for neutrophil-mediated inflammation. ATG5- or ATG7-deficient neutrophils reduce NADPH oxidase-mediated ROS production. In addition, the inhibition of NADPH oxidase reduces neutrophil degranulation [90] (Figure 2d,e).

## 5. Autophagy in Cancer Therapy—Targeting Autophagy

Tumor immunotherapy is a new generation of tumor treatment methods that rapidly develop after surgery, radiotherapy, chemotherapy and other traditional treatment methods. At present, there are many forms of tumor immunotherapy, which mainly include immune checkpoint inhibitors (ICIs), cancer vaccines and adoptive cellular immunotherapy. Autophagy has been found to be closely related to tumor immunotherapy and can either promote or inhibit tumor immune responses.

### 5.1. Autophagy Enhances the Effects of Immunotherapy

Autophagy is essential for the immunogenic release of ATP from dying cells. Chemotherapy-induced autophagy causes ATP release from mouse tumor cells, leading to the recruitment of immune cells to stimulate antitumor immune responses [91]. Autophagy defects inhibit T-cell-mediated killing in triple-negative breast cancer (TNBC). Defective autophagy leads to reduced Tenascin-C (TNC; an extracellular matrix glycoprotein) degradation, and high Tenascin-C expression is associated with poor prognosis in TNBC patients and negatively correlates with LC3 II expression and CD8^+^T cells [92].

### 5.2. Autophagy Attenuates the Effects of Immunotherapy

Hypoxia is a common feature of solid tumors [93]. It has been found that autophagy in a hypoxic environment can promote tumor immune escape. Hypoxia-induced resistance of lung tumor to CTLs is associated with autophagy [94]. Targeting beclin1 or Atg5 to inhibit autophagy leads to impaired pSTAT3 as well as SQSTM1/p62 accumulation and restores the susceptibility of hypoxic tumor cells to CTLs [95]. Hypoxia-induced autophagy in breast-cancer cells decreases susceptibility to NK-mediated lysis. Targeting beclin1 (BECN1) can inhibit autophagy and restore the level of Granzyme B in hypoxic cells and induce tumor regression by facilitating NK-mediated tumor-cell killing [96]. Therefore, the inhibition of autophagy can promote tumor immune responses.

### 5.3. Autophagy in Cancer Therapy

Taken together, autophagy activation acts as a double-edged sword in cancer initiation and progression. At present, a variety of autophagy regulators targeting different autolinks have been discovered for autophagy. mTOR inhibitors act as enhancers for autophagy initiation. The widely used rapamycin and its derivatives (everolimus, temsirolimus) form a complex with FKBP12, which changes the conformation of mTOR through binding to the FRB domain, dampening the kinase activity of the mTOR complex. 3-Methyladenine (3-MA) competitively binds to the ATP binding sites on the Class III PI3K kinase domain; therefore, it inhibits PI3K-initated autophagosome formation. Wortmannin serves the same purpose as a VPS34 inhibitor, to interfere with autophagy. Two other kinds of autophagy inhibitors, chloroquine (CQ) and hydroxychloroquine (HCQ), enter and accumulate in lysosomes and inhibit the degradation of cargos via the increase in pH to inactivate lysosomal enzymes. Monensin, as a lysosomotropic agent, directly changes the acidic environment in lysosomes. Bafilomycin A1, as a lysosomal vacuolar H+ ATPase (V-ATPase) blocker, restrains lysosomal acidification, thus inhibiting the fusion of autophagosomes and lysosomes (Table 1).

However, even though lysosomal inhibitors, along with 3-MA, are actively applied in clinical practice, there are still a number of pleiotropic pharmacologic effects. Bafilomycin A not only targets lysosomes but also affects V-ATPase on the plasma membrane and endosomes, interrupting the slightly alkaline pH of the cytosol, which can lead to acid-induced apoptosis. 3-MA and wortmannin simultaneously inactivate class I PI3-kinase, which interferes with the activity of downstream PKB, involved in the growth, proliferation and survival of target cells [101]. Hence, new insights for more selective drugs have opened up. SBI-0206965 is designed as a ULK1 kinase inhibitor and has been proved to have high selectivity without interfering with the FAK, mTOR and AKT signaling pathways. In vivo experiments of SBI-0206965 have demonstrated its effectiveness [102]. The exploration of other ULK1–ULK2-specific molecules is still flourishing, including MRT67307 and LYN-1604. VPS34-IN1 implements a specifically strong and rapid inhibition of PtdIns phosphorylation, while in vivo studies are dampened by solubility or metabolic issues [103]. Z-FA-FMK, as an agonist of Atg4B, has showed efficacy during autophagy both in vitro and in vivo [104]. Pyrazolopyrimidine sulfamates have been reported to selectively inhibit Atg7 and modulate autophagic processes including SQSTM1 aggregation and LC3II-complex formation [105].

In addition, the application of autophagy modulators has always faced the challenge of drug delivery with low bioavailability. This conundrum can hopefully be solved by encapsulating autophagy regulators into nanomaterials through nanomedicine approaches. Various nanoparticles (NPs) have been shown to induce autophagy, such as carbon-based NPs, polymeric nanoparticles, micelles, liposomes, etc. Cao et al., have found that hollow magnetic Fe3O4/graphene oxide (Fe3O4/GO) nanocomposites can effectively enhance the efficacy of rapamycin on hepatoma cells HepG2 [106]. Fan et al., have shown that the encapsulation of rapamycin in PLGA–PCL NPs (poly lactic-co-glycolic acid–polycaprolactone NPs) inhibited the proliferation of human breast-cancer cells and maintained and preserved the biological activity of rapamycin [107]. Chen et al., have demonstrated that rapamycin-loaded micelles have stronger antitumor effects than rapamycin alone in cancer cells HCT116 as well as in HELA cells [108]. Eloy et al., have found that co-loaded paclitaxel/rapamycin liposomes have greater lethality in mouse breast-cancer cells 4T1 [109].

Currently, as the only clinically approved autophagy inhibitors, clinical trials of CQ and HCQ alone or in combination with other therapies are ongoing for the treatment of cancer (Table 2).

The application of autophagy regulators in antitumor therapy has been gradually discovered, but there are still some problems. For example, autophagy plays different roles in different stages of cancer, inhibiting its progression in the early stage of tumorigenesis and promoting its progression in the late stage. It is worth thinking about how to select suitable autophagy regulators for combined therapy according to the different developmental stages of different tumors. In addition, the low bioavailability and delivery difficulties of autophagy regulators are also intractable issues. Research on nanoparticles as carriers of autophagy regulators is ongoing and is expected to further improve the utilization of autophagy-related drugs.

## 6. Conclusions

Autophagy is an important regulator of tumors. Autophagy affects innate and adaptive immunity through crosstalk with the tumor microenvironment and is critical for the generation, differentiation and maturation of immune cells. In clinical trials, autophagy modulators are gradually being used alone or in combination with other drugs in a variety of cancers. Therefore, the development of drugs targeting autophagy and how to balance the relationship between autophagy and the immune system are major challenges for future research. All figures were created with Biorender.com (accessed on 4 July 2022).

## Figures and Tables

**Table 1:** Autophagy modulators and their mechanisms.

Classification	Effect on Autophagy	Drugs	Mechanism	References
mTOR inhibitors	inducer	Rapamycin, Everolimus, Temsirolimus	Form a complex with FKBP-12 and inhibit mTORC1	[97]
Class III PI3K inhibitors	inhibitor	3-Methyladenine (3-MA), wortmannin	Inhibit autophagosome formation by inhibiting PI3K	[98]
Lysosomal inhibitors	inhibitor	Chloroquine (CQ)/Hydroxychloroquine(HCQ)	Increase the pH of the lysosome, cause the alkalization of the lumen and reduce its function; inhibit autophagic flux by reducing autophagosome–lysosome fusion	[99]
Lysosomal inhibitors	inhibitor	Bafilomycin A1	Inhibit both V-ATPase- dependent acidification and Ca-P60A/SERCA- dependent autophagosome–lysosome fusion	[100]

**Table 2:** Clinical trials targeting autophagy.

Drugs	Effect on Autophagy	Types of Cancer	Phase	Identifier
HCQ+Trametinib	inhibitor	Metastatic Neuroblastoma RAS (NRAS)	Not Applicable	NCT03979651
HCQ+Trametinib	inhibitor	Bile Tract Carcinoma (BTC)	Phase 2	NCT04566133
HCQ	inhibitor	Breast Cancer	Phase 2	NCT01292408
HCQ+Sorafenib	inhibitor	Hepatocellular Cancer	Phase 2	NCT03037437
HCQ+RAD001	inhibitor	Metastatic Clear Cell Renal Cell Carcinoma	Phase 1 Phase 2	NCT01510119
CQ	inhibitor	Small-Cell Lung Cancer (SCLC)	Phase 1	NCT00969306
HCQ+Cobimetinib +Atezolizumab	inhibitor	Gastrointestinal Cancer	Phase 1 Phase 2	NCT04214418
HCQ+ Paclitaxel +Carboplatin +Bevacizumab	inhibitor	Advanced/Recurrent Non-Small-Cell Lung Cancer	Phase 2	NCT01649947
HCQ+Abraxane +Gemcitabine	inhibitor	Advanced Adenocarcinoma Metastatic Adenocarcinoma	Phase 1 Phase 2	NCT01506973
HCQ+Etoposide +Mitoxantrone	inhibitor	Relapsed Acute Myelogenous Leukemia	Phase 1	NCT02631252
CQ+ Radiotherapy +Temozolomide	inhibitor	Glioblastoma Multiforme	Phase 1	NCT02378532
HCQ+Vorinostat +Regorafenib	inhibitor	Colorectal Cancer	Phase 2	NCT02316340

## Data Availability

Not applicable.
